# The distribution and evolution of Arabidopsis thaliana cis natural antisense transcripts

**DOI:** 10.1186/s12864-015-1587-0

**Published:** 2015-06-09

**Authors:** Johnathan Bouchard, Carlos Oliver, Paul M Harrison

**Affiliations:** Department of Biology, McGill University, Montreal, QC Canada

## Abstract

**Background:**

Natural antisense transcripts (NATs) are regulatory RNAs that contain sequence complementary to other RNAs, these other RNAs usually being messenger RNAs. In eukaryotic genomes, cis-NATs overlap the gene they complement.

**Results:**

Here, our goal is to analyze the distribution and evolutionary conservation of cis-NATs for a variety of available data sets for Arabidopsis thaliana, to gain insights into cis-NAT functional mechanisms and their significance. Cis-NATs derived from traditional sequencing are largely validated by other data sets, although different cis-NAT data sets have different prevalent cis-NAT topologies with respect to overlapping protein-coding genes. A. thaliana cis-NATs have substantial conservation (28-35% in the three substantive data sets analyzed) of expression in A. lyrata. We examined evolutionary sequence conservation at cis-NAT loci in Arabidopsis thaliana across nine sequenced Brassicaceae species (picked for optimal discernment of purifying selection), focussing on the parts of their sequences not overlapping protein-coding transcripts (dubbed ‘NOLPs’). We found significant NOLP sequence conservation for 28-34% NATs across different cis-NAT sets. This NAT NOLP sequence conservation *versus* A. lyrata is generally significantly correlated with conservation of expression. We discover a significant enrichment of transcription factor binding sites (as evidenced by CHIP-seq data) in NOLPs compared to randomly sampled near-gene NOLP-like DNA , that is linked to significant sequence conservation. Conversely, there is no such evidence for a general significant link between NOLPs and formation of small interfering RNAs (siRNAs), with the substantial majority of unique siRNAs arising from the overlapping portions of the *cis*-NATs.

**Conclusions:**

In aggregate, our results suggest that many cis-NAT NOLPs function in the regulation of conserved promoter/regulatory elements that they ‘over-hang’.

**Electronic supplementary material:**

The online version of this article (doi:10.1186/s12864-015-1587-0) contains supplementary material, which is available to authorized users.

## Background

Natural antisense transcripts (NATs) are a regulatory class of RNA that contains complementary RNA sequence to other RNAs, these other RNAs usually being messenger RNAs. NATs can bind partially to mRNAs through base complementarity, and can inhibit transcription or translation by different mechanisms; these include transcriptional interference [1], RNA masking [2], CpG methylation [3], and RNase-H-mediated mechanisms [4]. NATs can overlap their gene targets (where they are termed cis-NATs), or they can be located away from them (trans-NATs). The latter might be derived from transcribed pseudogenes [5].

Although NATs are still not fully understood, their role in development is being revealed as important. For example, in mammals, the expression of the brain-derived neurotrophic factor, which is a key factor in neuronal differentiation, growth, maturation, and maintenance, is upregulated when its endogenous cis-NAT is artificially degraded [6]. In comparison, although transgenic plants producing NATs have been used to study gene functions [7,8], comparatively less functional analysis has been done on endogenous plant NATs. Nonetheless, they are known to have important roles. For example, during vernalization, the floral repressor gene FLC is silenced transiently by its NAT [9,10]. Large-scale sequencing and microarray analysis indicates that the model plant Arabidopsis thaliana has thousands of cis-NATs [11-13]. A tiling-array analysis of the *A. thaliana* transcriptome under a variety of stress conditions (including high salinity, drought and cold stress), led to the discovery of several thousand cis-NATs [11]; their expression under these stress conditions required the expression of overlapping sense genes. A further tiling-array analysis revealed that many thousands of un-annotated cis-NATs were responsive to levels of the hormone abscissic acid in seeds [12]. A recent comprehensive analysis demonstrated the existence of thousands of cis-NATs in *A. thaliana*, using a variety of methods such as strand-specific RNASeq, quantitative RT-PCR and custom expression arrays [13]. Also, widespread occurrence of stress-responsive NATs, has been demonstrated in the rice *Oryza sativa* and other plants [14-17]. Several lines of evidence suggested that trans-NATs in rice can be made from pseudogenes and make regulatory small interfering RNAs [14]. Strand-specific RNA sequencing indicated that of over 2000 detected cis-NATs, >500 of them were associated with specific stress response conditions, such as salt, drought and cold stress [15]. Analysis of several hundred cis-NAT pairs implicated in stress reponse in rice and *A. thaliana,* demonstrated distinct distribution patterns for cis-NAT-derived small interfering RNAs [16].

Here, our goal is to analyze the distribution and evolutionary behaviour of cis-NATs of protein-coding genes in A. thaliana, to gain insights into their functional mechanisms and the significance thereof. What are the specific evolutionary trends (both in terms of sequence conservation and expression conservation) for these sequences Brassicaceae genomes? Do the parts of cis-NATs that do not overlap genes function in interference with transcription factor binding sites, or do they make small interfering RNAs? We find that significant conservation of cis-NAT sequence is correlated with conservation of expression in A. lyrata, and is linked to enrichments/depletions of other features (CHIP-seq peaks, small interfering RNAs, RNA structures, *etc.*). We discuss the functional significance of these observations. In terms of distribution, we observe a wide diversity of topologies for cis-NATs relative to neighbour protein-coding genes, both for cases with and without conserved regions in the non-overlapping parts (‘NOLPs’). However, cis-NAT data from different sources have differing prevalent topologies with respect to overlapping protein-coding genes, but, about half of the smaller set of curated cis-NAT annotations derived from conventional cDNA sequencing are validated by the other available data sets.

## Methods

### Data sets of transcribed sequences

Two-hundred and twenty-two cis-NATs were extracted from TAIR10 genome annotations [18]. These have full-length transcript evidence and lack ORFs. Conservation of transcription at cis-NAT loci in Arabidopsis lyrata was determined by genomic mapping of full-length transcripts against the A. lyrata genome, using BLAT [19]. The Okamoto2010 and Matsui2008 tiling-array-derived data sets are as described in refs. [12] and [13]. The RNASeq and RepTAS data sets derived in ref. [11] were also analyzed. They are the ‘Reproducibility-based Tiling-array Analysis Strategy’ and RNASeq data sets derived as described therein. These data were downloaded from the website http://chualab.rockefeller.edu/cgi-bin/gb2/gbrowse/arabidopsis/ [11].

Using Seqmap [20], *A. thaliana* small interfering RNA (siRNA) data were mapped against transcripts of protein-coding genes, then secondly also against the transcripts of TAIR cis-NATs, *or* (as appropriate) against genomic regions corresponding to the transcribed sequences of the other four cis-NAT data sets. We analyzed siRNAs that map uniquely in a forward direction on these NATs, and also, to label *trans-*acting cases, we assessed whether they map in reverse direction on the other transcribed regions.

### Genome alignments

To represent the diversity of the Brassicaceae, the genomes of 8 different species (Arabidopsis lyrata, Capsella rubella, Schrenkiella parvula, Leavenworthia alabamica, Sisymbirum irio, Brassica rapa, Aetheinema arabicum, and Eutrema salsugineum) were aligned against the TAIR9/10 A. thaliana genome assembly [21]. The relevant evolutionary tree is shown schematically in Additional file 1: Figure S1. The alignments were constructed as described in Haudry, et al. [21].

### Sequence conservation

**Figure 1:**
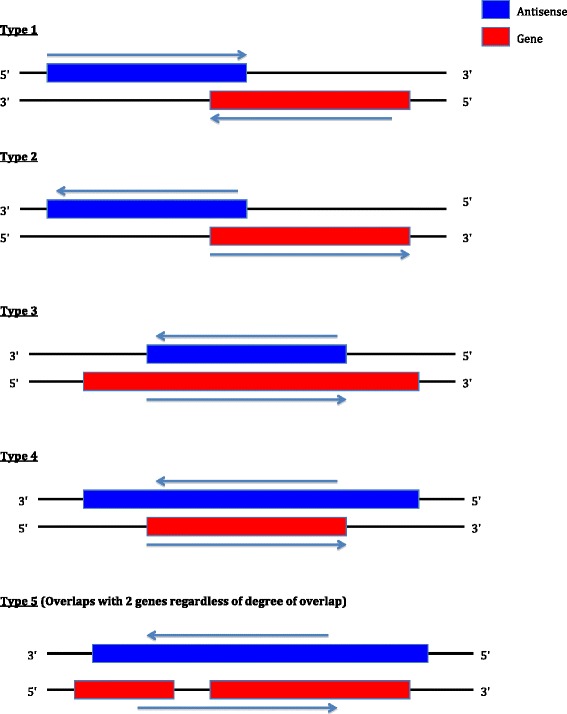
The different types of cis-NAT topology. There are five distinct topologies of cis-NATs and their anti-sense genes. They are depicted here schematically. Type 1 has divergent directions of transcription, whereas Type 2 has convergent directions.

The portions of cis-NATs that do not overlap protein-coding genes (termed ‘NOLPs’) were analyzed for significant conservation. Some cis-NATs (60 in total) are fully overlapped by a complementary gene (Figure 1, type 3) and were thus excluded from this. Sequence conservation was analyzed with phyloFit [22] and phyloP [23]. For PhyloFit, we used the neutral model (‘neutral.mod’) made by Haudry et al. [21], using the phylogenetic topology of nine Brassicaceae species [24,25] (Additional file 1: Figure S1), and the general reversible model of substitution that constrains substitution rates to maintain base frequencies over time. The phyloP program, using the original alignments and the neutral model from phyloFit, calculated conservation or acceleration P-values for the likelihood ratio test (LRT). Here, the likelihoods of observing the sequence under a neutral and non-neutral model are compared. A scale is calculated which is a coefficient that multiplies the neutral tree branches to get the non-neutral tree that produces the maximum likelihood. A scale =1 indicates neutrality because both trees overlap; a scale <1 or >1 indicates conservation or accelerated evolution, respectively. The variable D for the LRT is defined as:

$$ D=\hbox{--} 2 ln\left[ likelihood\ for\  null\  model/ likelihood\ for\  alternative\  model\right]. $$

Under the likelihood ratio theory, if the null model is true, D is a random variable with a chi-squared distribution (with a number of degrees of freedom given by the difference in number of parameters between the alternate and the null models). phyloP computes P-values, given D. Conserved cis-NATs have P-values <0.05 and scales <1. We applied the Holm-Bonferroni correction for multiple hypothesis testing for all tests for significant sequence conservation performed on the set of cis-NATs with genome alignments.

Since equivalent transcripts would occur in the different cis-NAT data sets we made a non-redundant list, to analyse the total population of cis-NATs. This non-redundant list (named ‘NR’) was compiled by sorting the total NOLP list (including TAIR cases) in decreasing order of sequence length, and then, for each case, progressively removing any overlapping NOLPs further down the list until no more can be removed.

### RNA secondary structure prediction

RNA secondary structure was predicted using RNAz [26], for multiple sequence alignments of NOLP conserved sequences. P-value thresholds of 0.5 and 0.9 were employed as recommended by the program authors.

### Random samples of near-gene NOLP-like genomic DNA 

For certain calculations, we assessed whether trends observed are significant compared to randomly sampled ‘NOLP-like’ near-gene DNA. The observed data sets were compared for various properties (e.g., significant sequence conservation, RNA structures, etc.), to a distribution of 500 random samples of near-gene DNA, of the same distribution of sequence lengths and relative positioning with respect to neighbor genes, as in the original observed data set. Each randomly-sampled genomic DNA stretch is adjacent to a known gene and not overlapping any gene transcript.

## Results & discussion

The cis-NATs can be classified into five types of topology with respect to the genes that they overlap (Figure 1). For the TAIR (*T*he *A*rabidopsis *I*nformation *R*esource) set (Table 1), the most common topology is Type 2 (64/222, 29%), where the cis-NAT and the overlapped gene are in divergent orientation (Table 1). This orientation guarantees a high degree of transcriptional interference between the cis-NAT and overlapped gene. For the other two large tiling-array-derived data sets named Okamoto2010 and Matsui2008 (Table 2), which do not exhibit defined intron-exon structure, this orientation less usual, with Type 3 the most common. These differences highlight the advantage of comparing different data sets derived by different methods.

**Table 1:** Numbers of different TAIR cis-NATs for the different topology types*

**Type of cis-NAT (as in** **Figure** 1 **)**	**Total number for the topology type**	**Number that have significant conservation (P < 0.05)**
Type 1	52 (23%)	14 (25%)
Type 2	64 (29%)	20 (36%)
Type 3	60 (27%)	--
Type 4	33 (15%)	17 (31%)
Type 5	13 (6%)	4 (7%)
**Total**	222	55

*NATs are classified into Types according to the following precedence: Type 5 before Type 3 before Type 4 before Type 1 before Type 2.

**Table 2:** Numbers of different cis-NATs for the different topology types for the four other data sets *

**Type of cis-NAT (** ***as in*** **Figure** 1 **)**	**Matsui2008**		**Okamoto2010**		**RNASeq**		**RepTAS**	
	**Total number for the topology type**	**Number that have significant conservation (P < 0.05)**	**Total number for the topology type**	**Number that have significant conservation (P < 0.05)**	**Total number for the topology type**	**Number that have significant conservation (P < 0.05)**	**Total number for the topology type**	**Number that have significant conservation (P < 0.05)**
Type 1	1246 (18%)	476 (47%)	665 (13%)	245 (56%)	4 (29%)	0	37 (54%)	18 (95%)
Type 2	605 (9%)	121 (12%)	433 (8%)	71 (16%)	4 (29%)	1	9 (13%)	1 (5%)
Type 3	3467 (51%)	--	3561 (68%)	--	6 (43%)	--	23 (33%)	--
Type 4	324 (5%)	142 (14%)	138 (3%)	39 (9%)	0	0	0	0
Type 5	1216 (18%)	283 (28%)	442 (8%)	82 (19%)	0	0	0	0
Total	6858	1022	5239	437	14	1	69	19

*NATs are classified into Types according to the following precedence: Type 5 before Type 3 before Type 4 before Type 1 before Type 2.

The smaller TAIR data set is a curated set of cis-NAT annotations derived from older Sanger sequencing techniques. A substantial fraction of these TAIR cis-NAT annotations are validated by the other data sets (labelled in Additional file 2). Overall, 88/162 TAIR annotations (54%) are validated by the other four data sets, and 24/55 of the cases with NOLP conservation (44%).

After classifying *A. thaliana* cis-NATs topologies in this way, we then examined conservation of expression and of non-gene-overlapping sequence in cis-NATs, to gain insight into the functional mechanisms of cis-NATs, and their significance. Specifically, we analysed: *(a)* conservation of expression of *A. thaliana* cis-NATs, in *A. lyrata*; *(b)* sequence conservation of non-overlapping parts (‘NOLPs’) of cis-NATs; *(c)* RNA structure prediction for these NOLP regions; *(d)* cross-referencing of cis-NAT expression conservation and NOLP sequence conservation with the occurrence of CHIP peaks, small interfering RNAs, transposons and protein homology.

### Transcription evidence in A. lyrata and other genomes

Is the transcription of A. thaliana cis-NATs conserved in A. lyrata? We examined expression at the loci of genes orthologous to those that overlap cis-NATs, in A. lyrata. A summary of analysis of conservation of expression in *A. lyrata* is arrayed in Table 3 (see footnote for details). A substantial fraction of cis-NATs, regardless of data set origin, have conservation of non-coding anti-sense transcription (28-35%) (Table 3).

**Table 3:** **Summary of conservation of expression, and of NOLP sequence conservation**

**Data set**	Conserved sequence*	Conserved anti-sense transcription in A. lyrata**	Conserved sequence and anti-sense transcription*
TAIR set (total 162)	12	57 (35%)	4
	55 ¶ (34%)		22
Matsui2008 set (total 3172)	889	1014 (32%)	453 ††
	1023 (32%)		350 †
Okamoto2010 set (total 1538)	314	435 (28%)	168 ††
	437 (28%)		131
Complete non-redundant NR set (total 4177)	584	1314 (31%)	191
	1323 (32%)		427

*The first value is for conserved sequence in A. lyrata and is calculated by pairwise BLASTN alignment (e-value <1e-10 [28]) adjacent to orthologous genes (determined using the bi-directional best hits method applied to the encoded protein sequences). The second value is for overall significant conservation across the nine species as determined by phyloP. Overall significant conservation is calculated as in Table 1.

**Anti-sense transcripts in A. lyrata have no ORF >100 codons and no protein homology in their own sense direction.

† P = 0.03, significant association of transcription and conservation, using hypergeometric test.

†† P ≤ 1x10^−25^, very significant association of transcription and conservation, using hypergeometric test.

¶ The total number of conserved are significantly depleted compared to randomly sampled near-gene DNA (P = 0.002, normal statistics). To assess this, for each of the three actual data sets listed, 500 samples of near-gene DNA of the same distribution of sizes and position relative to neighbour genes as the actual set were submitted to PhyloP calculation (as described in the *Methods*).

### Sequence conservation

*cis*-NATs conserved across several different Brassicaceae genera may be under selection pressure because they have important roles since the last common ancestor of these species. We searched for Arabidopsis thaliana cis-NAT loci that have significant sequence conservation across nine Brassicaceae species, including Brassica rapa, which is an important food crop. This was analyzed using PhyloP (as described in Methods) for the parts of cis-NATs that do not overlap protein-coding gene exons (i.e., ‘NOLPs’). We restricted our analysis to these parts, since we cannot de-convolute the conservation signals for the protein-coding exons for the other areas.

For the TAIR set of cis-NATs, we found that 55/162 (34%) of the cis-NATs had NOLPs with significant conservation (P < 0.05) (or 32/162 [20%] if we apply a Holm-Bonferroni [HB] correction) (Table 1 and Additional file 2). A small fraction, 7/162 (4%) have two sequence regions with significant conservation. For comparison, 199/232 (86%) of the protein-coding genes overlapped by the cis-NATs are significantly conserved for the same genome alignments.

As above for the total TAIR set, Type 2 is also the most common topology among those with significant conservation in Brassicaceae (Table 1), although Types 1 and 4 also arise for >25% of cases. For the other four cis-NAT data sets, Type 1 is very dominant as the most common amongst those with significantly conserved NOLPs (Table 2). For the two large tiling-array-derived NAT data sets, Matsui2008 and Okamoto2010, Type 1 comprises about half of all the significantly conserved cases. For Type 1 orientation, conserved NOLP cases would be able to overlap conserved promoter elements for the complementary gene; this is investigated below through analysis of positions of CHIP-seq peaks.

To assess the overall conservation statistics of all of the cis-NATs (TAIR set plus the four other data sets), we derived a non-redundant list of 4,177 cases (see Methods). Overall, comparable numbers are significantly conserved as for the TAIR set (1323/4177 [29%] or 743/4177 [18%] after HB correction).

In general, there is significant NOLP sequence conservation for 28-34% of cis-NATs (analysis summarized in Table 3). For the smaller TAIR set but not the two larger data sets, the NOLPs are significantly less conserved than randomly sampled near-gene DNA (P = 0.003 by normal statistics, see footnote, Table 3). There is a very significant correlation between sequence conservation of cis-NAT NOLPs in A. lyrata and conservation of transcription within A. lyrata, for the two largest data sets (Okamoto2010 and Matsui2008), but not the TAIR or NR sets (Table 3). This is probably because our method of constructing the NR list of cis-NATs is biased towards picking longer cases, and the TAIR set is too small in number to get a significant result. With one marginal exception (the Matsui2008 data set), there is no significant correlation between A. lyrata expression conservation and sequence conservation across the Brassicaceae (Table 3). This is evidence that in general this sequence conservation is not for maintenance of the cis-NATs NOLPs per se, but for other elements that are overlapped by them.

### RNA structure predictions for the significantly conserved cis-NATs

One strong indicator of the functional conservation of RNA molecules is the occurrence of conserved RNA secondary structure across diverse genome species. We applied the RNAz program [26] to search cis-NATs for RNA secondary structure that would indicate such functional significance. For the TAIR data set, rather a lot (12/55, 22%) have significant RNA secondary structures (RNAz P > 0.5), with 5/55 predicted with high probability (P > 0.9) (Table 4). These RNA structures may be linked to transcriptional interference, but could also have distinct functional roles. Interestingly, half of these TAIR cis-NATs with significant RNA secondary structures (RNAz P > 0.5) in their NOLPs are cases whose sequence conservation becomes non-significant upon correcting for multiple hypothesis testing with the HB method. Also, three of the NOLPs with significant RNA structures overlap pseudogenes that are adjacent to the anti-sense protein-coding genes, indicating that the RNA structures may have been formed from these pseudogenes.

**Table 4:** Numbers of different cis-NATs with RNA structures*

	**P > 0.5**	P > 0.5 **	**P > 0.8**	**P > 0.9**
TAIR data set	12/55 (22%)	6/55 (11%)	7/55 (13%)	5/55 (9%)
	2/55 (4%) ¶,	2/55 (4%)	1/55 (2%)	1/55 (2%)
Matsui2008	71/1365 (5%) ¶	--	46/1365 (3%)	32/1365 (2%)
Okamoto2010	22/485 (5%) ¶	--	13/485 (3%)	10/485 (2%)

* The fractions are of the totals that have significant sequence conservation. For the TAIR data, the first row is for RNAz calculations across the whole NOLP sequence. For non-TAIR data and for the second row for the TAIR data, these are cases that overlap CNSs (conserved non-coding sequences) identified in Haudry, et al. [21].

** Removing non-significantly conserved cases after Holm-Bonferroni correction.

¶ The total numbers of conserved are significantly enriched compared to randomly sampled near-gene DNA (P = 0.046 for the TAIR set, P < 0.00001 for the other two sets, normal statistics). To assess this, for each of the three actual data sets listed, 500 samples of near-gene DNA of the same distribution of sizes and position relative to neighbour genes as the actual set were generated (as described in the *Methods*).

In addition, for all three data sets (Table 4), we checked for RNA predictions that co-occur with previously defined conserved non-coding sequences (CNSs) [21]. In these cases, we find predicted RNA structures occurring in ~4-5% of all cis-NATs with such CNSs, significantly more than would be expected randomly (Table 4 footnote).

### Cross-referencing cis-NAT genomic loci with other genomic features

We cross-referenced the positions of NATs with the positions of various other entities in the genome: (i) CHIP-seq peaks; (ii) siRNA mappings; (iii) protein homology; (iv) transposons. This was to answer the question: is the NOLP sequence and expression conservation significantly correlated with the presence/absence of any of these other genomic features? Does this give us any insights into the functional mechanisms of cis-NATs?

#### CHIP-seq peaks

*cis-*NAT expression conservation in *A. lyrata* is only linked to enrichment of CHIP-seq peaks in the largest data set, Matsui 2008 (Table 5). However, *cis-*NAT sequence conservation in NOLPs is highly significantly linked to enrichment of transcription factor binding CHIP-seq peaks (from the PRI-CAT database [27]), indicative of the presence of transcription factor binding sites (Table 5). Furthermore, there is a very significant enrichment of such peaks in all three data sets, compared to randomly sampled near-gene DNA (Table 5 footnote).

**Table 5:** CHIP-seq peaks in cis-NATs *

	**# overlapping CHIP seq peaks**	**# of overlapping CHIP seq peaks in cases with significant sequence conservation**	**# of overlapping CHIP seq peaks in cases with conservation of expression in A. lyrata**
TAIR data set	209 ¶	81	87
Matsui2008	2916 ¶	999 ††	1014 †
Okamoto2010	1060 ¶	377 †††	313

* Counts are for individual non-identical CHIP-seq peaks. Significance of enrichment of CHIP-seq peaks using hypergeometric probability:

†, P = 0.02 (non-significant if a HB correction is applied, see Table 3), †† P = 0.001, ††† P < 0.0001.

¶ These total numbers of CHIP-seq peaks are highly significantly enriched compared to randomly sampled near-gene DNA (P < 0.00001, normal statistics). To assess this, for each of the three actual data sets listed, 500 samples of near-gene DNA of the same distribution of sizes and position relative to neighbour genes as the actual set were generated (as described in the *Methods*).

#### Small interfering RNA (siRNA) mappings

siRNA mappings (described in Methods) were cross-referenced with the cis-NAT NOLPs and OLPs (i.e., gene ‘*O*ver*L*apping *R*egions’) (Table 6). For the large Matsui2008 and Okamoto2010 data sets, the number of unique siRNA mappings to the cis-NATs that are trans-acting, is a small fraction of the total. Thus, most siRNA production can be assumed to be cis-acting. The substantial majority arises from OLPs (Table 6). Also, for cis-NAT NOLPs generally, or just for those with sequence conservation across Brassicaceae, there is a significant associated depletion of siRNA mappings (Table 6). This indicates that such sequence conservation is not due to siRNA production. Also, for the TAIR set, siRNAs in NOLPs are significantly depleted for cases that have conserved expression in *A. lyrata*, furthermore indicating that these NOLPs are not conserved to make siRNAs (Table 6).

**Table 6:** Occurrence in NOLPs of siRNAs*

**Data set**	**Total number of unique siRNA mappings (NOLP)**	**Total number of unique siRNA mappings (OLP)**	Number of unique trans-acting siRNA mappings** (NOLP)	**# of unique siRNAs in NOLPs (significant sequence conservation)**	**# of unique siRNAs in NOLPs (conservation of expression in** ***A.lyrata*** **)**
TAIR	579 ¶	701	4	101 †	32 †
Matsui2008	51283 ¶	78879	4609	12565 †	13750 †
Okamoto2010	23965 ¶	64804	2112	3949 †	4490 †

*These counts are for non-redundant lists across all siRNA data sets. OLP stands for ‘*O*ver*L*apping *P*art’.

**Calculated as described in Methods.

*Significant depletions using hypergeometric probability: † P < 0.0001.

¶ These total numbers of unique siRNA mappings are highly significantly *depleted* compared to what arises in randomly sampled inter-gene DNA (P < 0.00001 using normal statistics). To assess this, for each of the three actual data sets listed, 500 samples of near-gene DNA of the same distribution of sizes and position relative to neighbour genes as the actual set were generated (as described in the *Methods*). TAIR: 0.0001 . Okamoto: 0.0003 Matsui <0.00001.

#### Protein homology

A very small fraction of NOLP sequence contains any protein sequence homology (0-3%), whether there is significant sequence conservation or not (Table 7). This indicates that the results are not affected by the presence of undetected protein-coding exons.

**Table 7:** **Occurrence of transposons and protein homology in cis-NATs**

**Data set**	Amount of NOLP genomic DNA containing transposons for significantly conserved NOLPs versus total NOLPs*	Amount of NOLP genomic DNA containing protein homology for significantly conserved NOLPs versus total NOLPs**
TAIR	0 (0%) (4479, 5%)	7 (0%) (2701, 3%)
Matsui2008	63323 (12%) (432754, 26%)	13855 (3%) (42349, 3%)
Okamoto2010	18433 (10%) (154126, 26%)	1406 (1%) (4256, 1%)

*Transposon details taken from TAIR10 annotations.

**Protein homology to Arabidopsis annotated proteins (BLASTP [21] e-value ≤0.0001).

#### Transposons

The amount of NOLP sequence containing transposons is hugely depleted in cases with significant sequence conservation (Table 7), reducing from 26% to 10-12% of the total genomic DNA in the NOLPs, for the Okamoto2010 and Matsui2008 data sets. Such a substantial reduction in transposon occurrence is what would be expected for regions under selection not to accept sequence insertions. There is also a depletion of transposons for the TAIR set, but not as much (5% to 0%). This may be due to how the TAIR cis-NATs are defined or curated, with some transposon-derived cases being designated transposon-related genes.

## Conclusions

Taken in aggregate, our results suggest that cis-NAT NOLPs may function in regulation of promoter/regulatory elements in the *Arabidopsis* clade. Such NOLPs are significantly enriched for transcription factor binding sites as determined by CHIP data, compared to randomly sampled ‘NOLP-like’ DNA; also significantly conserved NOLP cases have more transcription factor binding sites than unconserved cases. The substantial conservation of cis-NAT expression in A. lyrata, is correlated with significant sequence conservation in the cis-NAT ‘NOLPs’ for comparison to this species; however, generally there is no correlation with significant sequence conservation across Brassicaceae. This cross-Brassicaceae sequence conservation is linked to enrichment of CHIP-seq peaks, and depletion of siRNAs in the NOLPs. Most siRNA production can be assumed to be *cis-*acting, and the substantial majority arises from OLPs (‘*O*ver*L*apping *P*arts’), rather than NOLPs. However, significant enrichment of RNA structures in the NOLPs indicates that there may be conservation of transcribed functional elements in the cis-NATs NOLPs per se.

Thus, promoter/regulatory elements conserved across Brassicaceae may be modulated in a specific clade (here *Arabidopsis*), by a form of transcriptional interference from a ‘over-hanging’ cis-NAT NOLP that has been formed relatively recently in evolution, in the last common ancestor for the clade (in this case the Arabidopsis genus).

Generally we observe many points of agreement between the different data sets analyzed, across the diverse calculations performed. Indeed, although cis-NAT data from TAIR and from tiling-array data have differing prevalent topologies with respect to overlapping protein-coding genes, encouragingly, most (~54%) curated TAIR cis-NAT annotations are validated by the other available data sets.

There are some limitations to this bioinformatics analysis. Larger data sets of small interfering RNAs from a greater variety of *A. thaliana* plant tissues would give us a clearer picture of their derivation from cis-NATs. Interpretation of these results is limited by our lack of appropriate expression data for other species, which would give use more insight into the processes of NAT evolution, and into the degree of clade specificity of the phenomena observed. Also, improved quality of the genome assemblies of the other *Brassicaceae* would also, of course, be beneficial.

## Additional files

Additional file 1: Figure S1. Evolutionary tree for the species used for conservation analysis. This is a schematicized version of the evolutionary tree (derived in Haudry, et al. [21]) that was used for conservation analysis calculations. The species are picked to sample two main lineages of *Brassicaceae* (plus *A. arabicum* as a more distant relative of *A. thaliana*) that are expected to maximize detection of purifying selection (represented by yellow/green and orange coloration) *C. papaya* is the outgroup used to root the *Brassicaceae* tree. Additional file 2: List of TAIR cis-NATs with significant NOLP sequence conservation.
