# Supplementary figures and images for: The distribution and evolution of Arabidopsis thaliana cis natural antisense transcripts
Source: BMC Genomics. 2015 Jun 9;16(1):444. doi: 10.1186/s12864-015-1587-0 (PMC4467840; doi:10.1186/s12864-015-1587-0)

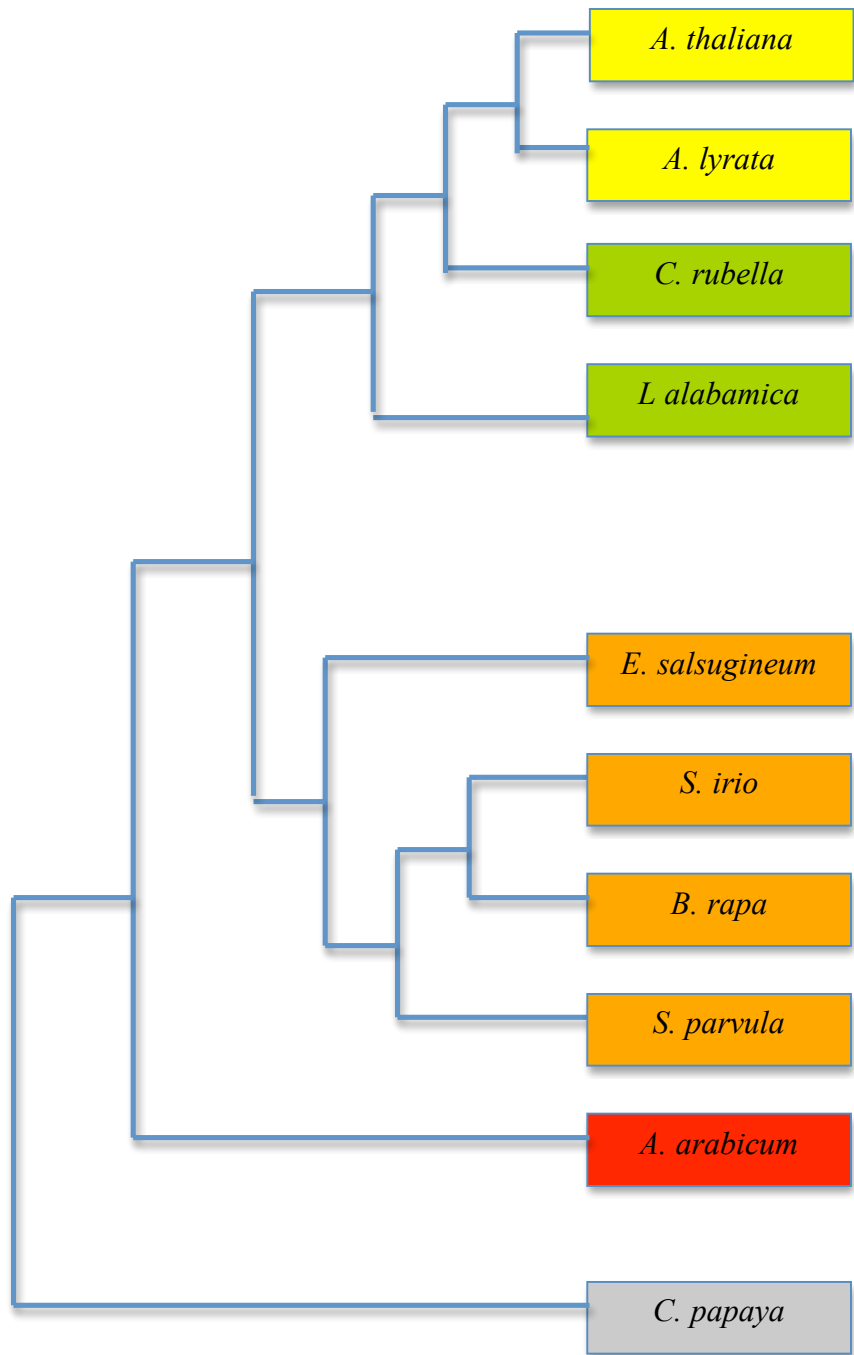

Supplement: Additional file 1: Figure S1. — Evolutionary tree for the species used for conservation analysis. This is a schematicized version of the evolutionary tree (derived in Haudry, et al. [21]) that was used for conservation analysis calculations. The species are picked to sample two main lineages of Brassicaceae (plus A. arabicum as a more distant relative of A. thaliana) that are expected to maximize detection of purifying selection (represented by yellow/green and orange coloration) C. papaya is the outgroup used to root the Brassicaceae tree. [file 12864_2015_1587_MOESM1_ESM.pdf]
